# Comparison of a practice-based versus theory-based training program for conducting vacuum-assisted deliveries: a randomized-controlled trial

**DOI:** 10.1007/s00404-021-06159-8

**Published:** 2021-08-07

**Authors:** Julian Marschalek, Lorenz Kuessel, Maria Stammler-Safar, Herbert Kiss, Johannes Ott, Heinrich Husslein

**Affiliations:** Department of Obstetrics and Gynecology, Medical University of Vienna, Spitalgasse 23, 1090 Vienna, Austria

**Keywords:** Objective structured assessment of technical skills (OSATS), Simulation, Training, Vacuum-assisted delivery, Vacuum extraction

## Abstract

**Purpose:**

Vacuum-assisted deliveries (VAD) are complex procedures that require training and experience to be performed proficiently. We aimed to evaluate if a more resource intensive practice-based training program for conducting VAD is more efficient compared to a purely theory-based training program, with respect to immediate training effects and persistence of skills 4–8 weeks after the initial training.

**Methods:**

In this randomized-controlled study conducted in maternity staff, participants performed a simulated low-cavity non-rotational vacuum delivery before (baseline test) and immediately after the training (first post-training test) as well as 4–8 weeks thereafter (second post-training test). The study’s primary endpoint was to compare training effectiveness between the two study groups using a validated objective structured assessment of technical skills (OSATS) rating scale.

**Results:**

Sixty-two participants were randomized to either the theory-based group (*n* = 31) or the practice-based group (*n* = 31). Total global and specific OSATS scores, as well as distance of cup application to the flexion point improved significantly from baseline test to the first post-training test in both groups (*p*_all_ < 0.007). Skill deterioration after 4–8 weeks was only found in the theory-based group, whereas skills remained stable in the practice-based group.

**Conclusion:**

A practice-based training program for conducting VAD results in comparable immediate improvement of skills compared to a theory-based training program, but the retention of skills 4–8 weeks after training is superior in a practice-based program. Future studies need to evaluate, whether VAD simulation training improves maternal and neonatal outcome after VAD.

**Supplementary Information:**

The online version contains supplementary material available at 10.1007/s00404-021-06159-8.

## Introduction

Operative vaginal delivery (OVD) with the aim to expedite delivery and thereby reduce maternal and fetal morbidity is an essential skill for obstetricians. The rates of OVD vary worldwide and lie between 3 and 15% [1, 2]. In the last decades, OVD decreased in countries like the United States and Sweden, whereas it increased in countries like Austria or Norway. The choice of instrument (i.e. forceps or vacuum) varies widely around the world, with forceps used in up to 16% in primipara in the U.K. compared to less than 0.5% in Austria and Sweden.

OVDs are complex procedures that require a combination of fine motor skills, understanding of the maternal and fetal anatomy and the mechanics of vaginal birth and therefore need training and experience to be performed proficiently. However, the need for an OVD occurs mostly unscheduled and under stressful circumstances, often caused by maternal or fetal distress. Although the use of a vacuum extractor has been proven to be effective, feasible and safe, its use can provoke significant maternal and fetal morbidity [3–7]. Failed OVD has been directly linked to increased fetal and maternal morbidity and is often attributed to the skill level of the operator [6, 8–10]. The reported rate of failed vacuum-assisted deliveries (VAD) varies throughout the literature and lies between 1 and 34% [3, 11–14].

The traditional approach to teach VAD can be described as “learning-by-doing-model” or a “see one, do one-approach” under supervision of an expert. However, this approach is no longer appropriate for medical professionals and bears limitations as learning under stressful conditions limits the effectiveness of the educational and learning process [15, 16]. Acute situations withdraw the timespan to perform tactile trainings or to reconsider and modify the conceptualization. Therefore, theoretical guidelines, training models, and simulators have been evaluated [17–22]. Simulation training has been tested in many fields of medical education and has been shown to improve performance in real clinical situations, especially in procedures that occur rarely or in high-stress environments, making it a potentially ideal modality for VAD training [23, 24]. Nonetheless, due to diminished working hours for trainees and because instructed hands-on-training demands more time and staff resources, theory- and video-based training methods have increasingly aroused interest [17, 21–23].

A recent study tested both models on medical students with no previous exposure to VAD and could demonstrate a significantly higher improvement of VAD skills in the practice-based hands-on-training [17]. However, we do not know whether this is also true in maternity staff with previous exposure to VAD or already skilled in VAD; a key question in efforts aiming to improve the quality of care in obstetric clinical routine.

In this randomized-controlled study conducted in maternity staff, we aimed to evaluate if a practice-based training program for conducting VAD is more efficient compared to a purely theory-based training program, using a validated procedure-specific skill rating scale [25]. Moreover, we intended to measure the persistence of skills 4–8 weeks after the initial training.

## Materials and methods

This randomized-controlled trial was carried out at the Department of Obstetrics and Gynecology, Medical University of Vienna, Austria, between April 2017 and February 2020.

Maternity staff (obstetricians, residents and midwives) were invited to participate in this study.

Oral and written informed consent was obtained from all participants. There were no specific inclusion or exclusion criteria. After enrollment, a unique study identification number was assigned to each participant and participants were randomized into one of the following two groups: (1) a theory-based VAD training program and (2) a theory and practice-based VAD training program. Randomization was carried out by means of serially numbered sealed envelopes, according to a computer-generated randomization plan using a one-to-one randomization. Baseline demographic data, such as age, gender, training status (depending on previously performed VAD), number of previously performed VAD training programs and number of attended vaginal deliveries, were collected.

After randomization, all participants performed a low-cavity non-rotational vacuum delivery in a simulated environment, which served as the baseline test. For all simulation scenarios, the Lucy and Lucy’s Mum instrumental delivery birth simulator (Paradigm Medical Systems, Portland, OR) and the Kiwi® Omnicup vacuum extractor (Clinical Innovations, LLC., Putzbrunn, Germany) were used.

After the baseline test, participants allocated to group 1 underwent a 30-min power-point training session including instructions how to perform a VAD on basis of the technique described by Aldo Vacca [26], followed by a self-guided hands-on training for 15 min. Participants allocated to group 2 underwent the same 30-min power-point training session including the same instructions how to perform a VAD, followed by a one-to-one-instructed hands-on training for 15 min. After the training, every participant performed a second low-cavity non-rotational vacuum delivery on the birth simulator, which served as the first post-training test.

Four to eight weeks after the initial training program, a third low-cavity non-rotational vacuum delivery was performed to determine the persistence of skills (= second post-training test).

All simulated vacuum deliveries were recorded using a handheld camera. Participants were filmed in a way, that their identity was not recognizable (i.e., without the participants head). Furthermore, the participants’ voices were altered using a software program (Wondershare Filmora 9, Wondershare Software Co., Ltd.).

**Fig. 1:**
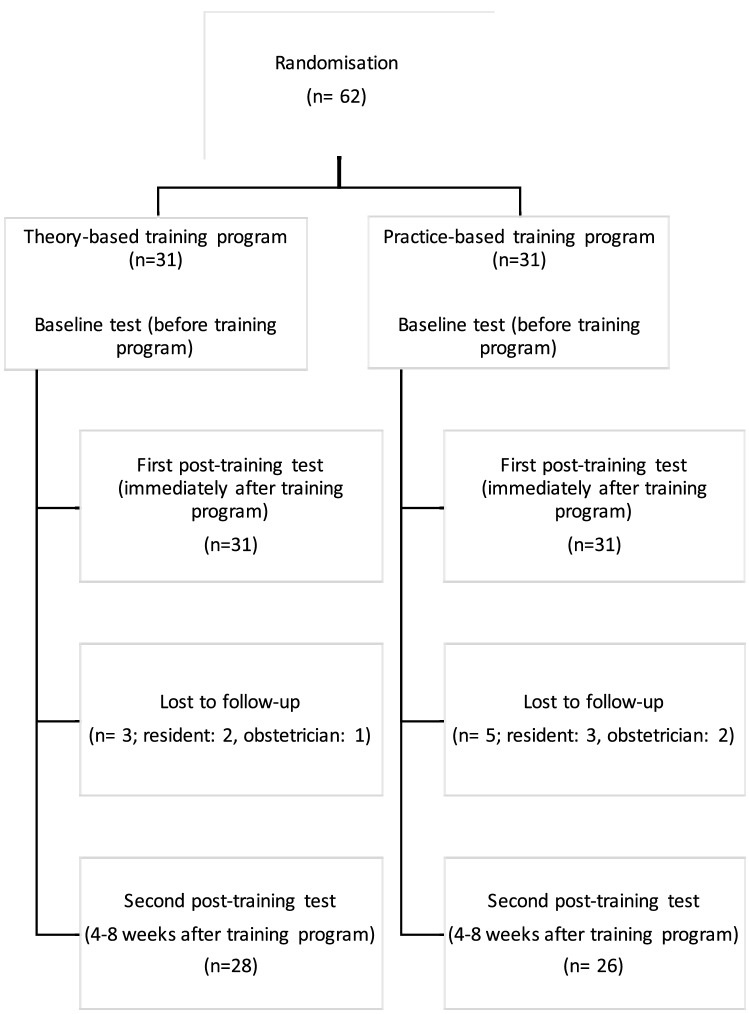
Consort diagram of the progress through the study

After having performed all three video-recorded procedures, all participants had the possibility to join a practice-based training program for the first time/or again, depending on the prior randomization. A Consort diagram of the progress through the study is shown in Fig. 1.

The study’s primary endpoint was to compare training effectiveness between the two study groups by assessing the videos of the baseline test and the first post-training test of a simulated low-cavity non-rotational vacuum delivery using a validated global and procedure-specific skill rating scale, which was developed on basis of an objective structured assessment of technical skills (OSATS) rating scale [25]. Assessments of the video-recordings were carried out by an obstetrician with 15 years of experience and expert status in conducting vacuum-assisted deliveries. The rater was blinded to the trainee’s identity, allocation, experience status as well as the number and order of the video-recordings.

Secondary endpoints included the persistence of training effect and the correct application of the vacuum cup by measuring the distance of the cup application to the flexion point at the time of the actual simulations.

With a sample size of *n* = 30 subjects per group, we were able to exclude a reasonable difference of total scores (effect size: *d* = 0.8) with a power of 80% and a type 1 error rate of 5% (two-sided), providing an asymptotic relative efficiency for the Wilcoxon–Mann–Whitney test of 0.864 (worst case) as compared to the parametric alternative [27].

The study was conducted in accordance with the Declaration of Helsinki and was registered with Clinical-Trials.gov (NCT03111498). Since the participation was voluntary, the need for an ethical approval for this study was waived by the Ethics Committee of the Medical University of Vienna.

## Results

Sixty-two participants were recruited and randomized to either the theory-based group (*n* = 31) or the practice-based group (*n* = 31). Three participants in the theory-based (and five participants in the practice-based group were lost to follow-up after the first post-training (Fig. 1). There were no significant differences between groups concerning demographic characteristics (Table 1).

**Table 1:** Demographic characteristics of the study participants

	Theory-based training (*n* = 31)	Practice-based training (*n* = 31)	*p *Value
Age (years)	27 (24–34)	29 (25–36)	0.302
Sex			1.0
Female	22 (71)	22 (71)	
Male	9 (29)	9 (29)	
Employees’ Status			1.0
Resident	20 (64.5)	21 (67.7)	
Obstetrician	9 (29)	9 (29)	
Midwife	2 (6.5)	1 (3.2)	
Attended vaginal births	35 (6–200)	19 (1–250)	0.463
Previous exposure to VAD	14 (45.2)	12 (38.7)	0.797
Number of previously performed VAD	0 (0–8)	0 (0–10)	0.987
Previous Trainings in VAD			0.344
Yes	12 (38.7)	9 (29)	
No	19 (61.3)	22 (71)	

Categorical data are presented as the frequency and percentage. Continuous variables are expressed as the median and interquartile range (IQR)

### Baseline test

There was no difference between the total global and specific OSATS scores between groups. Furthermore, there was no difference regarding the distance of cup application to the flexion point (Supplement Table).

### Immediate training effects

Total global and specific OSATS scores, as well as distance of cup application to the flexion point improved significantly from baseline test to the first post-training test in both groups (Table 2). Improvements were comparable between groups (Table 3).

**Table 2:** Developing of cup application and OSATS scores in the theory—and the practice-based group

	Distance to Flexion Point^*^	*p*-Value_0-1_^a^	*p*-Value_1-2_^b^	*p*-Value_0-2_^c^	Total Global Rating Score^#^		*p*-Value_0-1_^a^	*p*-Value_1-2_^b^	*p*-Value_0-2_^c^	Total Specific Rating Score^§^	*p*-Value_0-1_^a^	*p*-Value_1-2_^b^	*p*- Value_0-2_^c^
*Theory*-*based* *Training* (*n* = 31)													
Baseline test	2 (1–2)	0.000			12 (10–17)		0.000			38 (36–45)	0.000		
1st Post-training test	0 (0–1)		0.004		19 (16–21)			0.001		56 (48–63)		0.001	
2nd Post-training test	2 (0–2)			0.262	14.5 (12.25–17.5)				0.373	47 (43–50)			0.016
*Practice*-*based* *Training* (*n* = 31)													
Baseline test	2 (1.5–3)	0.000			13 (9–16)		0.002			40 (34–49)	0.007		
1st Post-training test	0 (0–1)		0.197		19 (15–20)			0.403		57 (41–61)		0.703	
2nd Post-training test	0 (0–1)			0.000	17.5 (13.75–19)				0.006	53.5 (45–56.5)			0.000

Continuous variables are expressed as the median and interquartile range (IQR)

*Distance to Flexion point – distance from cup application to flexion point in centimeters (cm)

^#^Total Global Rating Score – maximum points to achieve 25

^§^Total Specific Rating Score – maximum points to achieve 80

^a^*p*-value_0-1_ – difference between Baseline test and First post-training test

^b^*p*-value_1-2_ – difference between First and Second post-training test

^c^*p*-value_0-2_ – difference between Baseline test and Second post-training test

**Table 3:** Comparing the relative difference (delta, Δ) of OSATS scores and cup application between the theory- and the practice-based groups

	Theory-based training	Practice-based training	*p *Value
Δ-*Baseline vs. First post-training test*			
Distance to Flexion Point	− 1 (− 2–0)	− 2 (− 2 to − 1)	0.137
Total Global Rating Scale	6 (1–9)	5 (2–9)	0.972
Total Specific Rating Scale	10 (5–22)	14 (7–22)	0.994
Δ-*First vs. Second post-training test*			
Distance to Flexion Point	1 (0–2)	0 (0–1)	0.028
Total Global Rating Scale	− 4.5 (− 6.75– − 1.25)	− 1 (− 5.25–3)	0.039
Total Specific Rating Scale	− 8 (− 15– − 0.25)	− 2.5 (− 15–12)	0.082

Continuous variables are expressed as the median and interquartile range (IQR)

### Long-term training effects

For evaluation of long-term training effects, baseline tests were compared to the second post-training test after 4–8 weeks. In the practice-based training group, global and specific OSATS scores, as well as distance of cup application to the flexion point were still significantly improved compared to baseline test, whereas in the theory-based practice group, only the specific OSATS scores were still better compared to the baseline test (Table 2).

To demonstrate deterioration of skills over time, we further compared the first (immediate) and second (4–8 weeks after baseline) post-training tests between groups (Table 2). In the practice-based training group, global and specific OSATS scores, as well as distance of cup application to the flexion point remained comparable over time (Table 2). In contrast, in the theory-based practice group, global and specific OSATS scores, as well as distance of cup application to the flexion all deteriorated significantly over time (Table 2). Additionally, we compared the relative difference (delta) of OSATS scores and distance of cup application to the flexion point, revealing a significantly greater deterioration over time in the theory-based group compared to the practice-based group (Table 3).

To determine which procedure-specific skills decreased at first after the training, we compared the procedure-specific ratings scores directly after the training with those 4–8 weeks thereafter in the two groups. In the theory-based group, there was a significant decrease with respect to “vaginal examination for the presenting part, rotation and station” (First post-training test: 4 (IQR 3–5) vs. Second post-training test: 3 (IQR 2–4), *p* = 0.002), “assessment of the need for oxytocin” (First post-training test: 1 (IQR 1–5) vs. Second post-training test: 1 (IQR 1–1), *p* = 0.022), “Applies the cup on the flexion point” (First post-training test: 5 (IQR 5–5) vs. Second post-training test: 3.5 (IQR 1.25–5), *p* = 0.002) and “direction of traction follows the pelvic curve” (First post-training test: 5 (IQR 4–5) vs. Second post-training test: 4 (IQR 2.25–5), *p* = 0.010). Analysis of the practice-based group revealed only a significant worsening in “protection of the perineum” (First post-training test: 5 (IQR 1–5) vs. Second post-training test: 1 (IQR 1–5), *p* = 0.006).

## Discussion

The results of our randomized-controlled trial show that a practice-based training program for conducting VAD results in comparable immediate improvement of skills compared to a theory-based training program, but that retention of skills 4–8 weeks after training is superior in a practice-based program.

Hands-on models have already been shown to significantly enhance the operator’s technical skills [23, 24], but so far only few studies evaluating different training programs in VAD exist.

One study including 36 participants reported that a simulation-based training program for VAD resulted in a significant improvement in the accuracy of cup application and theoretical knowledge [28]. Unfortunately, no validated global or procedure-specific skill rating scale was used and no long-term follow-up was performed.

Hilal and colleagues performed a randomized-controlled study comparing a hands-on training versus a video demonstration in medical students with no previous exposure to VAD [17]. They reported a significant improvement in OSATS scores in the hands-on training group immediately after the training and in a second simulation 4-days later. In contrast to our study, they used a self-developed 40-item OSATS scoring system, which was validated in the same study, demonstrating some construct validity. Furthermore, all study participants were medical students with no previous exposure to VAD. Therefore, no baseline test was performed, limiting the information value of the results.

In this study, we have addressed two critical points that, to the best of our knowledge, have not been studied so far. First, whether maternity staff with experience in conducting VAD also show greater improvement from a hands-on training program and second, whether a hands-on training program has a longer-term effect on VAD skills compared to a purely theoretical training program.

Based on our results, both, the theory- and the practice-based training programs lead to significant improvement of global and specific OSATS scores immediately after the training, independently of the trainees’ experience status. But only in the practice group, these skills remained 4–8 weeks after the initial training. This long-term effect was also seen when looking at correct cup application: cup application in the second post-training test was significantly more accurate in the practice-based group than in the theory-based group. Of note, to place the cup over the flexion point is a crucial factor for a successful VAD [29, 30]. Correct cup placement is particularly important in mal-positions of the fetal head [26, 31], is reported to reduce “pop-offs” and minimize the duration of the procedure [32, 33]. In contrast, an incorrect cup placement is reported to increase the risk of failed VAD [34] and to contribute to the severity of fetal head traumata, such as cephalohematoma or subgaleal hemorrhage [35, 36].

In the theory-based group, OSATS scores of procedure-specific skills decreased after 4–8 weeks in the categories “vaginal examination”, “cup application”, “direction of traction” as well as “assessment of the need for oxytocin”. In the practice-based group, only the category “protection of the perineum” worsened. Specific technical skills, such as correct cup placement over the flexion point and direction of traction, are crucial to reduce the risk of failed VAD and therefore seem to be especially important to retain as long as possible after training [26, 34].

To the best of our knowledge, our study is the first randomized-controlled trial evaluating different VAD training programs conducted in maternity staff reporting on long-term effects assessed by validated OSATS scores. But our study has limitations: first, our study does not provide evidence that the improved skills in a simulation setting translate to an improved maternal and neonatal outcome after VAD. Studies demonstrating transfer of skills from a simulation to a clinical setting are limited [24], but one study reported decreased rates of cervical, severe labial, or high vaginal lacerations as well as less neonatal scalp and facial injury after simulation training in OVD [37]. Second, in the theory-based training program, we used a standardized power-point training session and a single live tutor for the instructions how to perform a VAD. This approach can be seen as a limitation due to inter-individual variations or possible differences in the quality of the daily teaching performance. Third, our follow-up period of 4–8 weeks cannot rule out that repetitive trainings might be useful for a long-term persistence of the trainings effect. Of note, a 2012 study evaluating the retention of skills in the management of obstetric complications reported that residents maintained the trainings effect for a year and that repeating the simulation after one year brought additional improvement [38]. This is in accordance with professional societies and guidelines recommending hands-on simulation training of obstetric complications on a yearly basis [19, 39, 40]. If and how a repeated VAD hands-on training proves beneficial one year after the initial training has to be evaluated in future studies.

In conclusion, this study shows that a practice-based training program for conducting VAD results in a prolonged training effect compared to a theory-based training. Especially procedure-specific skills, such as correct cup placement over the flexion point and direction of traction remained stable 4–8 weeks after the hands-on training, whereas they deteriorated after the theory-based training. The immediate training effects were comparable between the practice-based and theory-based training program. Future studies need to evaluate, whether VAD simulation training improves maternal and neonatal outcome after VAD.

## Supplementary Information

Below is the link to the electronic supplementary material.

Supplementary file1 (DOCX 15 kb)

## Data Availability

The datasets used and analyzed during the current study are available from the corresponding author on reasonable request.
