# Supplementary material for: Comparison of a practice-based versus theory-based training program for conducting vacuum-assisted deliveries: a randomized-controlled trial
Source: Arch Gynecol Obstet. 2021 Aug 7;305(2):365–72. doi: 10.1007/s00404-021-06159-8 (PMC8840931; doi:10.1007/s00404-021-06159-8)
Supplement: Supplementary file 1 — Supplementary file1 (DOCX 15 kb) [file 404_2021_6159_MOESM1_ESM.docx]

**Supplement Table.** Comparing cup application and OSATS scores between the theory- and the practice-based group before the training program

|  | **Theory-based Training**  **(n=31)** | **Practice-based Training**  **(n=31)** | **p-Value** |
| --- | --- | --- | --- |
| **Baseline test** |  |  |  |
| Distance to Flexion Point | 2 (1–2) | 2 (1.88 –3) | 0.268 |
| Total Global Rating Scale | 12 (10–17) | 13 (9–16) | 0.735 |
| Total Specific Rating Scale | 38 (36–45) | 40 (34–49) | 0.994 |
|  |  |  |  |

Continuous variables are expressed as the median and interquartile range (IQR).
